# Hemicellulose-based hydrogels for advanced applications

**DOI:** 10.3389/fbioe.2022.1110004

**Published:** 2023-01-09

**Authors:** Ying Xu, Kun Liu, Yanfan Yang, Min-Seok Kim, Chan-Ho Lee, Rui Zhang, Ting Xu, Sun-Eun Choi, Chuanling Si

**Affiliations:** ^1^ Tianjin Key Laboratory of Pulp and Paper, Tianjin University of Science and Technology, Tianjin, China; ^2^ Department of Forest Biomaterials Engineering, College of Forest and Environmental Sciences, Kangwon National University, Chuncheon, South Korea; ^3^ Department of Finance, Tianjin University of Science and Technology, Tianjin, China; ^4^ State Key Laboratory of Tree Genetics and Breeding, Northeast Forestry University, Harbin, China

**Keywords:** hemicellulose, hydrogel, biomedical material, adsorption, sensor

## Abstract

Hemicellulose-based hydrogels are three-dimensional networked hydrophilic polymer with high water retention, good biocompatibility, and mechanical properties, which have attracted much attention in the field of soft materials. Herein, recent advances and developments in hemicellulose-based hydrogels were reviewed. The preparation method, formation mechanism and properties of hemicellulose-based hydrogels were introduced from the aspects of chemical cross-linking and physical cross-linking. The differences of different initiation systems such as light, enzymes, microwave radiation, and glow discharge electrolytic plasma were summarized. The advanced applications and developments of hemicellulose-based hydrogels in the fields of controlled drug release, wound dressings, high-efficiency adsorption, and sensors were summarized. Finally, the challenges faced in the field of hemicellulose-based hydrogels were summarized and prospected.

## 1 Introduction

Hydrogels are polymer materials with three-dimensional spatial network structure, which are usually formed by physical or chemical crosslinking of long chain hydrophilic polymers. Physical cross-linking method refers to the formation of cross-linking structure between polymer chains through winding or various weak interactions such as hydrogen bonds or ionic bonds, without the formation of new chemical bonds. The chemical cross-linking method refers to the formation of new covalent bonds, the formation of structural units through copolymerization or condensation reaction of hydrogel, its structure is irreversible, heating will not change its structure (Hoffman, 2012). Compared with the physical cross-linking method, the chemical cross-linking hydrogel has better stability, and the cross-linking density is also controllable, so the physical properties of the hydrogel can be adjusted freely (Mondal et al., 2020). The high cross-linking degree of hydrogels enables them to have good water absorption and retention capacity, and will not dissolve after absorbing a large amount of water (Warren et al., 2017). Due to the differences in raw materials and preparation methods, hydrogels can be endowed with different physical and chemical properties and have been widely used in industrial adsorption (Baghbadorani et al., 2019), medical treatment (Hassan et al., 2018; Liu et al., 2020a; Wang H. et al., 2021; Deng et al., 2022), agriculture (Cheng et al., 2018; Wei et al., 2019), sensor (Lu et al., 2020; Suneetha et al., 2021) and other fields.

Hydrogels can be divided into natural polymer-based hydrogels and synthetic polymer-based hydrogels according to the source of the polymer. Although natural polymer including polysaccharides, gelatin, proteins and nucleic acid hydrogels have good biocompatibility, they are unstable and easy to be degraded (Shi et al., 2016). The composition, structure, and strength of synthetic polymer hydrogels such as polyacrylic acid, polyacrylamide, acrylic acid, methacrylic acid, N-isopropyl acrylamide, N-vinyl-2-pyrrolidone, *etc.* Are stable and controllable, but their biocompatibility is poor (Martínez-Gómez et al., 2017; Kesharwani et al., 2021). Therefore, the combination of natural polymers and synthetic polymers to prepare hydrogels will be a major development trend (Du et al., 2016; Yang et al., 2021). Hydrogels will have the advantages of both natural polymers and synthetic polymers, showing higher service life, gel strength and water absorption capacity, and more importantly, good biocompatibility.

**FIGURE 1:**
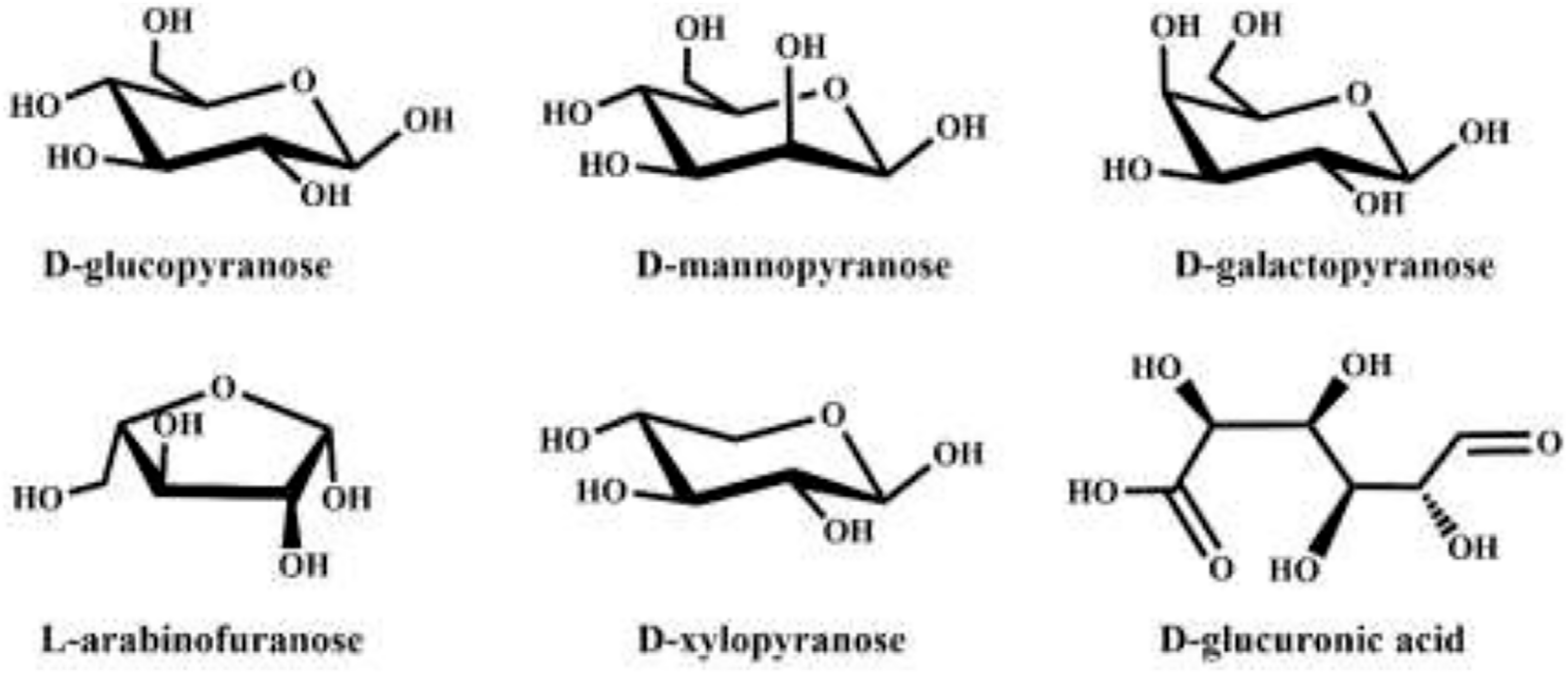
The monosaccharide composition of hemicellulose reproduced from: Kong et al. (2018), Springer.

Lignocellulose is the most abundant natural polymer on the earth, which has the advantages of extensive sources and renewable (Si et al., 2008; Si et al., 2013; An et al., 2019; Liu et al., 2021e; Huang et al., 2021; Qian et al., 2023). It is widely used in energy storage (Liu et al., 2021b; Xu et al., 2021b; Liu et al., 2021c; Liu et al., 2021f; Liu et al., 2022b; Du et al., 2022; Li et al., 2022; Xu et al., 2022), electromagnetic shielding (Liu et al., 2022d; Liu et al., 2022e), biomedicine (Du et al., 2019; Liu et al., 2020c; Xiong et al., 2021a; Xiong et al., 2021b; Ha et al., 2021), sensing (Lu et al., 2019; Ma et al., 2021; Liu et al., 2022a), antibacterial (Du et al., 2021a; Du et al., 2021b) and many other fields (Dai et al., 2017; Xie et al., 2019; Yang et al., 2019; Chen et al., 2020a; An et al., 2020; Wang H. et al., 2020a; Chen et al., 2020b; Liu et al., 2020b; Dai et al., 2020; Xu et al., 2021a; Zhang et al., 2021a; Liu et al., 2021g; Wang H. et al., 2021; Wang et al., 2022a; Liu et al., 2022c; Liu et al., 2022f; Liu et al., 2023). At present, there are many physical and chemical methods to extract lignocellulose from various raw materials, such as ultrasonic, grinding, enzymatic hydrolysis, acid treatment and alkali treatment (Chen et al., 2016; Liu et al., 2017a; Hu et al., 2017; Dai et al., 2018; Xie et al., 2018; Li et al., 2019; Xu et al., 2020a; Xu et al., 2020b; Li X. et al., 2020; Liu et al., 2021d; Zhang et al., 2021d; Liu et al., 2022g). Hemicellulose is a kind of heteroglycan, mainly composed of five-carbon sugars and six-carbon sugars, including xylose, galactose, arabinose, mannose, *etc.* (Figure 1). Hemicellulose, together with cellulose and lignin, constitutes plant cells and is the main component of lignocellulose, which is characterized by high degree of branching and low degree of polymerization (Feng et al., 2012). Hemicellulose has attracted much attention due to its low cost, good biocompatibility, and wide sources. Hemicellulose is rich in oxygen-containing groups, such as hydroxyl, acetyl and carboxyl groups and so on, which can be modified by etherification (Sun et al., 2019), esterification (Wang et al., 2017), graft copolymerization (Farhat et al., 2018) and other means. Thus, it can be used to synthesize hydrogels with excellent properties (Li et al., 2014). With the deepening of the research on hemicellulose structure, the preparation methods and application range of hemicellulose hydrogels are also expanding. In this paper, the preparation methods, and advantages of hemicellulose hydrogels as well as the advanced application fields and prospects are summarized, hoping to provide a reference for the research and development of new hemicellulose hydrogels in the future.

## 2 Preparation of hemicellulose-based hydrogels

### 2.1 Chemically cross-linking hydrogel

Chemical cross-linking refers to the method of forming covalent bonds between polymeric monomers, which makes the polymers bond more closely and thus have stronger stability and mechanical properties than physical cross-linking (Table 1).

**TABLE 1:** Fabrication methods and their advantages.

Methods	Characteristic	References
Physical crosslinking by hydrogen bond, ionic interaction, or hydrophobic interaction	Hypotoxicity, weak strength and stability	Zhang et al. (2021b)
IPN or semi-IPN	High strength, self-healing property	Li N. et al. (2020)
Chemical cross-linking by cross-linkers	Facile and common	Liu et al. (2022h)
Photoinitiated	Simple and efficient	Gao et al. (2015)
Enzymatic	Excellent biocompatibility	Markstedt et al. (2017)
Microwave irradiation	Rapid response, no hysteresis effect and good selectivity	Meena et al. (2014)
Glow discharge plasma	Environmentally friendly, easy to operate, low cost, small environmental pollution	Zhang et al. (2015)

#### 2.2.1 Radical polymerization

Free radical polymerization refers to the process in which monomers produce free radicals under the action of light, heat, radiation or initiator, and form polymer through a series of copolymerization. Polymers can be cross-linked by adding cross-linking agents, such as N,N′-methylenebisacrylamide (MBA) or glutaraldehyde, and so on (Peng et al., 2012). Chen T. et al. (2020) isolated hemicellulose from eucalyptus alkaline peroxide mechanical pulping (APMP) waste liquor. Hydrogen atoms on the hydroxyl group of xylan were captured in the initiator system of anhydrous sodium sulfite (Na_2_SO_3_) and ammonium persulfate (APS) to generate free radicals. With the addition of acrylic acid (AA), acrylamide (AM) and the cross-linking agent (MBA), the unsaturated double bond on MBA molecule is opened, and the hydroxyl group on the polymer is cross-linked, and the hydrogel with excellent water absorption and water retention is prepared by free radical polymerization. Seera et al. (2021) mixed xylan and gelatin in a certain proportion and used ethylene glycol diglycidyl ether (EDGE) as cross-linking agent to prepare hydrogel. The two epoxy rings of EGDE cross-linker were opened in a high alkaline medium of 1.5 M NaOH solution to form covalent bonds with the hydroxyl moieties of xylan and the amine moieties of gelatin. The opening of epoxy rings and the formation of covalent bonds will happen simultaneously so that the hydrogel can be cross-linked successfully.

Konjac glucomannan (KGM) has excellent film-forming ability, good biocompatibility, biodegradability, and gel-forming properties. However, its poor pH sensitivity behavior and strong water absorption limit its application (Chen et al., 2018; Wu et al., 2018; Wang et al., 2019a). The carboxyl modification of KG introduced carboxymethyl group, which reduced the intermolecular hydrogen bond and water absorption and could be used to prepare hydrogels (Xiao et al., 2015). Iron ion can also be used as a crosslinking agent in the preparation of hemicellulose hydrogels. Therefore, Wang et al. (2020b) prepared a novel hydrogel based on Dopamine–carboxymethyl konjac glucomannan (DCKGM) and L-Cysteine–carboxymethyl konjac glucomannan (CCKGM) with Fe^3+^ as cross-linker. The stable construction of the hydrogel is based on covalent coordination bonds between Fe^3+^ and catechol, and hydrogen bonding between DCKGM, CCKGM, and Fe^3+^. Due to the strong interaction of Fe^3+^ with DCKGM and CCKGM, the resultant is adherent, injectable, formable and pH sensitive. Furthermore, the hydrogel shows great potential in various applications (e.g., active compounds delivery) because of the ability to load EGCG.

The modification of hemicellulose can endow it with new functional groups and improve the polymerization degree, water solubility and thermal stability of hemicellulose. Thus, the properties of hemicellulose hydrogel can be improved or endowed, and its application scope can be expanded. Carboxymethyl hemicelluloses, the derivatives of hemicelluloses, have better solubility in water than hemicelluloses, and have hydroxyl and carboxyl functional groups as well (Ren et al., 2008).

Liu et al. (2022h) prepared carboxymethyl xylan (CMX) by mixing hemicellulose, NaOH and sodium monochloroacetate (SMCA) through etherification reaction. Then dopamine was grafted onto carboxymethyl xylan, and the resulting dopamine-grafted carboxymethyl xylan was denoted as CMX-DA. And AM, initiator APS and cross-linker MBA were added to prepare xylan-based hydrogels through free radical polymerization. APS could not only polymerize AM into PAM, but also capture hydrogen atoms from the hydroxyl group of CMX-DA to generate free radicals as the active site, so that PAM could be grafted onto the CMX chain and finally fabricate hydrogel. In addition, the catechol of dopamine imparted nanocomposite hydrogels with adhesion properties. Li et al. (2021) build a plant catechol-metal ion autocatalytic system composed of sodium lignosulfonate/iron ion (SL/Fe^3+^), and prepared a transparent hydrogel with elastic, electrical conductivity and UV blocking properties by grafting vinyl monomer onto carboxymethyl xylan (CMX). The introduction of Fe^3+^ could form coordination bonds with CMX to form DN structure during cross-linking, giving rise to improve mechanical performance (extension ratio: 460%, tensile stress: 23 kPa). Furthermore, the SL/Fe^3+^ system can activate KPS to generate a large number of free radicals, and rapidly initiate the graft copolymerization of AM on the carboxymethyl xylan (CMX) backbone at room temperature instead of heating, oxygen removal, or UV treatment for the preparation of multifunctional xylan hydrogels.

IA is a hydrophilic unsaturated organic acid with good biocompatibility. It can copolymerize and introduce carboxylic side groups into polymers that are able to form hydrogen bonds with corresponding groups. Liu et al. (2017b) prepared the hydrogels by free radical copolymerization from NIPAAm/IA mixtures as the monomers in the presence of APS and TEMDA as the initiator and acylated hemicellulose as the macromolecular cross-linker. The acylated hemicellulose chain, acting as framework, was grafted by NIPAAm or IA and was cross-linked with other acylated hemicellulose chains containing unsaturated double bonds, resulting in the formation of the hydrogels. PVA was used as an enhancer to produce the temperature- and pH-sensitive hydrogels and it also had good biocompatibility.

Photoinduced crosslinking has the advantages of no pollution, fast curing and low energy consumption in the gelling process. In the reaction process, photoinitiator is needed to trigger the formation of free radicals, which is an efficient and environmentally friendly cross-linking technology. Firstly, hemicellulose was esterified with maleic anhydride (MA) to prepare hemicellulose derivatives containing vinyl, which were cross-linked with unsaturated functional groups of NIPAAm under UV light to form gels (Yang et al., 2011). Gao et al. (2015) used MBA as cross-linker and 2, 2-dimethoxyphenylacetophenone as photoinitiator to graft and copolymerize xylan and glycidyl methacrylate-modified xylan (GMAX) with N-isopropylacrylamide and acrylamide respectively *via* ultraviolet irradiation. The hydrogels were prepared and the properties of hydrogels of two different materials were compared. This method has been shown to be a mild and efficient method with applications in hemicellulose-based hydrogels.

Radiation cross-linking refers to a method in which polymers can produce free radicals under the induction of high-energy rays such as γ-rays and electron beams, and thus polymerize to form three-dimensional networks. It has the advantages of rapid reaction, good selectivity, safety, non-toxic and mild conditions. It is a common method in the preparation of hydrogels by free radical polymerization (Wiesbrock et al., 2004; Fekete et al., 2016). Meena et al. (2014) mixed kappa-carrageenan (kC) with xylan, added water soluble sodium persulfate (KPS) as initiator, and then added polyvinylpyrrolidone (PVP) to get composite hydrogel with microwave radiation. The introduction of PVP can significantly improve the physicochemical and rheological properties of kC/Xylans, and also lead to the formation of semi-IPN structure, thus improved the gelling properties of the resulting hydrogels. It has been proven that the combination of KPS initiator and microwave irradiation can improve the incorporation rate of PVP.

Enzymatic cross-linking has the advantages of mild reaction conditions, simple operation, and good biocompatibility, which can not only avoid the toxicity of chemical cross-linking agents, but also improve mechanical strength. In recent years, its application in biomedicine has attracted more and more attention (Jafari et al., 2022; Wang et al., 2023). Kuzmenko et al. (2014) conjugated xylan from spruce to tyramine, and the conjugated compound was cross-linked under the initiation system of horseradish peroxidase (HRP) and hydrogen peroxide. The primary amine group in tyramine is coupled to the carboxyl group of glucuronic acid unit in xylan. Under the HRP/H_2_O_2_ initiation system, the two carbons in the ortho position of xylan-tyramine conjugate form C-C bond, and the C-O bond is formed between the ortho carbon atom and the oxygen atom of phenolic hydroxyl group, thus crosslinking into hydrogel in 20 ± 5 s at room temperature. This study reduced the amount of H_2_O_2_ in the initiation system and made the product have excellent biocompatibility. Markstedt et al. (2017) prepared hydrogels based on O-acetyl-galactoglucomannans (GGMs) from spruce functionalized with tyramine induced GGMs polymerization to form gels through HRP/H_2_O_2_ system. Due to the advantages of low cost, cell friendliness and adjustable mechanical properties, the hydrogel can be applied to biomedical materials.

In order to overcome the problems of chemical by-products, chemical residues and energy consumption, *etc.*, the glow discharge electrolytic plasma (GDEP) trigger system has been applied in the field of hemicellulose hydrogels due to its advantages such as mild and controllable reaction, simple device, low cost and no pollution (Zhang et al., 2015). Zhang et al. (2015) used hemicellulose as backbone, hydroxyl radicals which were produced by GDEP as initiators, acrylic acid (AA) and N-isopropyl acrylamide (NIPAAm) as monomers, N, N-methylene double acrylamide (MBA) as crosslinking agents to prepare temperature/pH dual sensitivity reed hemicellulose-based hydrogel. They prepared reed hemicellulose hydrogel for adsorption of heavy metal ions in aqueous solution. The results showed that discharge voltage and discharge time have important effects on the adsorption of hydrogel (Zhang et al., 2016).

Photoinitiation, radiation crosslinking and enzyme crosslinking are currently environmentally friendly, mild, and safe methods for hydrogel synthesis. However, these methods have not been fully developed due to some limitations. With the progress of science and technology in the future, the development of non-toxic, environmentally friendly crosslinking agents and mild, energy-saving crosslinking methods is an important research direction of hemicellulose-based hydrogels.

#### 2.2.2 Click chemistry

The concept of “click chemistry” was first proposed by Kolb et al. (2001) in the early 21st century, which is a method for synthesizing macromolecules based on carbon-heteroatom bonds. This simple and rapid reaction can avoid the limitations of traditional modification methods such as esterification and etherification. And it can provide a mild and efficient modification way for hemicellulose modification, endowing it with specific structure, properties and functions (Mamidyala and Finn, 2010; Meng and Edgar, 2016). Copper-catalyzed azide-alkyne cycloaddition (CuAAC) and thiol-ene/yne click reaction are representative reactions of click chemistry, which can be used for polymer modification (Hoyle and Bowman, 2010; Enomoto-Rogers and Iwata, 2012; Meng and Edgar, 2016). Compared with thiol-ene/yne click reaction, there are few reports on modification of hemicellulose by CuAAC reaction. There is still a lot of space for the development of future.

The thiol–ene reaction has gained much attention in chemical synthesis. The reaction can usually be performed under mild reaction conditions giving high conversion and selectivity, using water as solvent. In addition, no toxic metal catalysts are needed, making the use of thiol-ene chemistry tempting for the modification of polysaccharides. Thiols and amines are valuable functional groups having high reactivity. Pahimanolis et al. (2015) designed a simple method for functionalizing xylan with thiols, amines and amino acids by combining traditional etherification and thiol-ene reactions. Firstly, the hydroxyl group on xylan reacts with allyl chloride under alkaline conditions at 40°C to introduce the allyl group into the backbone of xylan, and then thiols are introduced into the backbone of xylan by click chemistry. This method provides a broad possibility for the development of new polysaccharide-based materials, and through thiol-thiol oxidative coupling, free thiol groups can be used to form hydrogels, and the shape of the resulting hydrogels can be well controlled, thus the fields of application were expanded. Maleki et al. (2017) designed a fully interpenetrating double network structure hydrogel from O-acetyl-galactoglucomannan (AcGGM), which was formed by the click reaction of thiol-ene and free radical polymerization. Firstly, the single-network hydrogels were prepared by grafting AA oligomeric chains from the unsaturated sites of AcGGM-Ma *via* free radical cross-linking. The cross-linking of the second network was then mediated through two separate pathways: thiol-ene IPN cross-linking (the thiol-ene click reaction between sulfhydryl functionalized AcGGM and polyethylene glycol diacrylate (PEG-DA)) and free-radical IPN cross-linking. While both strategies effectively afford IPNs, the route involving making the second network from thiolated AcGGM and PEG-da clearly affords IPNs with much higher shear moduli. In addition, the fraction of renewable material will be significantly higher in this case where both individual networks are based on AcGGM and from these perspectives the thiol-ene crosslinking strategy may be more viable and the resulting IPNs more suitable as membranes, absorbants and supports serving in more or less pressurized environments.

Wang et al. (2022b) propose an injectable nanocomposite hydrogel that prepared by light-induced thiol-ene addition between methacrylate modified O-acetyl-galactoglucomannan (GGMMA) and thiolated cellulose nanocrystal (CNC-SH). CNC-SH reinforced the GGMMA hydrogel as both a nanofiller and a crosslinker to GGMMA resulting in an interpenetrating network *via* thiol-ene addition. This light-induced thiol-ene cross-linking method is a mild and fast cross-linking method, the addition of thiol-ene makes the internal network of the hydrogel uniform, but also has good mechanical properties.

### 2.2 Physically cross-linking hydrogel

Physical cross-linking method refers to the formation of hydrogels using freezing, ultrasound, light and other methods. Hydrogen bonds, ionic bonds and intermolecular forces play a role in gelation. The hydrogel is greatly affected by the environment, and the size of holes in it cannot be adjusted easily (González et al., 2018). Although the strength and stability of hydrogels formed by physical cross-linking method are not as good as those formed by chemical cross-linking method, there is no need to add initiator, cross-linking agent or some organic solvents in the preparation process, so as to reduce the chemical toxicity and increase the application scope of hydrogels (Yan et al., 2010).

#### 2.2.1 Non-covalent forces

Gabrielii et al. (2000) separated xylan from poplar by alkali extraction combined with ultrafiltration and mixed it with chitosan. Through the electrostatic interaction between the microcrystalline domain formed by the combination of chitosan chain and xylan chain and the acid group of xylan and the amino group in chitosan, hemicellulose-chitosan hydrogel was prepared for the first time. Studies have shown that hydrogels can be formed when the content of chitosan is 5%–20%, and the swelling degree of hydrogels is positively correlated with the content of chitosan, and hydrogels dissolve when the content of chitosan exceeds 20%. Qi et al. (2015) grafted methylguanidine hydrochloride onto the backbone of xylose to prepare xylose modified by guanidine ion. Then, ethylene glycol was used as the crosslinking agent, and the modified xylose was entangled with the exfoliated layered anionic montmorillonite (MMT) clay nanosheet under the dispersion of sodium polyacrylate (PAAS) to form xylose-based hydrogel. The xylan-based hydrogel is connected by hydrogen bonds and shows intermolecular adsorption due to its internal sponge like porous structure, which has a rapid self-healing ability and shows good swelling performance. In addition, due to the addition of inorganic MMT, this xylan-based hydrogel has good heat resistance. Gabrielii et al. (2000) added chitosan into the xylan solution. The microcrystalline structure was formed between xylan and chitosan and served as a physical cross-linking point. The microcrystalline was connected to conduct the crystal arrangement of the polymer, forming a eutectic network structure. When the content of chitosan reaches 5%–20%, the co-crystalline structure forms a network structure, and the hemicellulose/chitosan composite hydrogel is prepared.

#### 2.2.2 Freeze-thaw cycle technique

Freeze-thaw technique is a physical cross-linking method that promotes the generation of hydrogen bonds in polymers through repeated freezing and thawing. As a hydrogel formation method, freeze-thaw method can not only cross-link polymers in solution, but also avoid the residue of cross-linker that can cause inflammation in human body (Liu et al., 2009). Zhang et al. (2021b) simply and efficiently prepared hemicellulose/polypyrrole (H/PPY) composite hydrogel by freeze-thaw technique. First, all reactants including H/PPY suspension, PVA, glycerol, and borax were initially mixed, and the solution was frozen at 20°C for 12 h to gelatinize and thawed at room temperature for 4 h. After two freeze–thaw cycles, the resultant hydrogels were soaked into NaCl solution. Borax was decomposed into B(OH)^3−^ and B(OH)^4-^ which could further form a variety of complexation and cross-linking with H/PPY, glycerol, and PVA. The strong hydrogen bonds among PVA, H/PPY complex, and glycerol promoted the formation of hydrogels. The hydrogel has good mechanical strength and can be stretched 250 times at 50% strain and still have good reproducibility.

Guan Y. et al. (2014) prepared hemicellulose, PVA and chitin nanowhiskers with compact structure, high strength and thermal stability hydrogels by freeze-thawing cycle. Because these polymers are rich in hydroxyl groups, hydrogen bonds are easily formed between them, and the hydrogen bond network is very stable at low temperatures. Therefore, during the freeze-thaw cycle, their molecular chains are closely bound by physical cross-linking and shows good mechanical properties. The chitin nanowhiskers embedded homogeneously in PVA/hemicellulose form more hydrogen bonds, which significantly improves the mechanical strength and thermal stability of hydrogel. And the hydrogel structure becomes stronger with the increasing times of freeze/thaw cycle. Multiple cycles make the polymer network more compact and reduce the space for water. After three freeze-thaw cycles, the hydrogel forms a layered structure and the swelling degree is reduced (Guan et al., 2015).

### 2.3 Composite hydrogel

As hemicellulose macromolecular structure has many branches and complex chemical composition, the relative interaction between molecules in hydrogels prepared from pure hemicellulose is weak, resulting in unsatisfactory mechanical strength (Liu et al., 2022h). However, hemicellulose and other kinds of polymers or nanoparticles can be combined to produce hemicellulose based composite hydrogels with different functions. For example, the combination of hemicellulose and chitosan can improve the swelling and mechanical properties of hydrogels, and can also give hydrogels the adsorption performance of heavy metal ions (Ayoub et al., 2013). The composite of nanocellulose and hemicellulose can enhance the toughness, viscosity and self-recovery properties of hydrogels (Karaaslan et al., 2011). In hemicellulose hydrogels, the introduction of chitin nanocrystalline whiskers with average length of 200 nm and width of 40 nm can improve the mechanical and thermal properties of hydrogels (Guan Z. et al., 2014).

Polypyrrole (PPY) has emerged as a polymer with the advantages of easy synthesis, high conductivity, stability and good biocompatibility, which can be introduced into hydrogels and applied to wearable strain sensors (Kenry, 2018). Zhang et al. (2021b) firstly, mixed hemicellulose white poplar and pyrrole monomer in water to form H/PY composite. FeCl_3_ was then added to induce the growth of the PPY chain. Afterwards it is then mixed with PVA, glycerol and borax and polymerized into hydrogels by strong hydrogen bonding. Glycerol can form a large number of hydrogen bonds with water molecules, which can greatly improve the water retention of hydrogel (71.8%). The introduction of glycerol and NaCl could inhibit the ice crystals in the hydrogels and reduce the freezing and thawing temperatures of the hydrogels to −43.1°C and −22.1°C. The conductivity of the hydrogel increases from 2.1 S/m to 5.1 S/m when PPY is introduced into the hemicellulose hydrogel. And the introduction of glycerol can improve the conductive path of hydrogel network.

Polyaniline (PANI) is a kind of polymer with attractive properties such as easy synthesis, environmental friendliness, electrical conductivity, and excellent biocompatibility. However, its application is limited because it is not degradable and cannot be dissolved in ordinary non-polar or even weakly polar organic solvents. To overcome this shortcoming, the use of aniline oligomers has been proposed instead of polymers, because they have similar electrical conductivity, in addition to good solubility and clear structure. Moreover, the degradation byproducts of oligomers can be taken up by macrophages and can subsequently undergo renal clearance to exit the body. Wen et al. (2020) prepared conductive hemicellulose hydrogels (CHHs) with tunable swelling behavior, controllable conductivity, and stable network structure by introducing carboxyl terminated aniline pentamer (CTAP) and epichlorohydrin into xylan-rich hemicellulose network through a facile one-pot approach. The equilibrium swelling ratio of the hydrogel can be adjusted by changing the content of CTAP and epichlorohydrin. Due to the hydrophobicity, thermal stability, and electrical conductivity of CTAP, when the content of CTAP increases, the water absorption of hydrogel decreases, the thermal stability increases, and the electrical conductivity increases to 2.11 × 10^–3^ S/m.

When hemicellulose is added to other polymer networks, it often acts as an enhancer (Prakobna et al., 2015) or cross-linker (Karaaslan et al., 2011). Berglund et al. (2020) investigated the influence of xylans and glucomannans in cellulose/hemicellulose composite hydrogel on the mechanical properties of hydrogel. The study showed that glucomannans improved the compressive elastic modulus of composite hydrogel, and xylans increased the elongation at break of composite hydrogel. Dax et al. (2016) prepared a green cross-linking agent based on O-acetyl galactoglucomannan. When hemicellulose is used as a cross-linking agent, the hydrogel has excellent swelling performance and can quickly absorb water 154 times its weight, but its mechanical strength is low. Adding NFC to the hydrogel can effectively improve the mechanical strength without affecting its adsorption performance. Gong et al. (2022a) modified hemicellulose nanoparticles (HC) with tannic acid (TA) and the composite hydrogels were formed by mixing acrylic acid with APS (initiator) and MBA (cross-linking agent) evenly and adding TA@HC nanoparticles as nanometer filler. Then the hydrogel was immersed in AlCl_3_ solution and Al^3+^ was introduced to obtain PAA-TA@HC-Al^3+^ hydrogel. The hydrogels were multi-network structures of covalent bonds and non-common bonds, which can improve the mechanical properties of hydrogels. It was also found that the hydrogel had good electrical conductivity, toughness and antimicrobial properties. The cross-linking mechanisms of Al^3+^ in hydrogel structures are Al^3+^-TA@HC-carboxylate hybrid coordination and Al^3+^-TA@HC coordination, and Al^3+^-carboxylate coordination. In addition, the hydrogel has antioxidant and UV resistance properties.

The introduction of inorganic particles into hydrogels has been shown to improve the strength and toughness of hydrogels (Lei et al., 2019). Nanomaterials such as calcium phosphate nanoparticles (Nie et al., 2019), bentonite (Liu et al., 2021a), metal nanoparticles (Arvizo et al., 2012) or graphene (Mohammadrezaei et al., 2018) can also give gels a variety of properties, enabling them to have a wide range of applications in many fields (Han et al., 2020).

Bentonite is a natural non-metallic mineral with montmorillonite as main component. It has the structure of two-dimensional nanomaterials and has the characteristics of expandability, hydrophilicity, stability, non-toxicity, and adsorption (Feng et al., 2004). Liu et al. (2022h) introduced bentonite into hydrogels prepared by cross-linking dopamine grafted carboxymethyl xylan with PAM. The exfoliation of bentonite would improve the compatibility between inorganic phase and organic phase. The bentonite formed physical interaction with polymer chain. The PAM chains could be integrated with the neighboring bentonite sheets by a mutual combination of polymer chains, and they could be cross-linked through non-covalent bonding such as hydrogen bonding and polymer chain entanglement, resulting in a nanocomposite hydrogel 3D network formation. With the introduction of bentonite, the Young’s modulus of the hydrogel increased to 1,449.3 kpa, indicating that the addition of bentonite could enhance the cross-linking density and improve the mechanical strength of the hydrogel.

The combination of iron oxide nanoparticles (Fe_3_O_4_) and polysaccharide hydrogels can be used as magnetic field response hydrogels due to their large magnetic moment, excellent superparamagnetization and high stability in water medium. Zhang and Sun (2020) added Fe_3_O_4_ nanoparticles to acrylic acid (AAC), then added hemicellulose and cross-linking agent, and formed semi-interpenetrating magnetic composite hydrogel by using H_2_O_2_-VC initiated system. The redox initiator system H_2_O_2_-Vc generated oxygen anion radical (O^2-^) which initiated the polymerization of acrylic acid, and modified Fe_3_O_4_ nanoparticles joined into this polymerization by covalent bonds through the vinyl group on the surface. The introduction of Fe_3_O_4_ can not only increase the crosslinking density of the hydrogel, but also give the hydrogel superparamagnetic property, and the magnetization intensity increased with an increase in the content of Fe_3_O_4_ nanoparticles. The HC/PAAc semi-IPN magnetic hydrogel showed high adsorption capacity and smart swelling property in lysozyme adsorption experiments, which has potential applications in drug delivery and magnetic separation.

Graphene oxide (GO) and reduced graphene oxide (RGO) have gained wide attention due to their potential role in enhancing cellular response and modulating protein adsorption and cell behavior. Graphene and its derivatives contain reactive oxygen functional groups, making the surface easy to form covalent, electrostatic and hydrogen bonds with biomolecules (polypeptides, nucleic acids and growth factors). Ali et al. (2022) introduced graphene oxide/reduced graphene oxide (GO/RGO) as nano-filler into hemicellulose/chitosan/nano-hydroxyapatite (HAp) composite hydrogel scaffold. The fillers are uniformly distributed in the hydrogel, forming a porous three-dimensional network with good mechanical properties. The addition of GO/RGO can improve the mineralization tendency of apatite and increase the osteogenic capacity of the composite. Further studies showed that the composite hydrogel has cellular activity and protein affinity, which is helpful for bone repair and regeneration, and is a potential material for bone tissue engineering.

Due to the low molecular weight and heterogeneity of hemicellulose, the mechanical strength of the composite hydrogel is limited. The introduction of interpenetrating polymeric network (IPN) or semi-interpenetrating polymeric network (semi-IPN) structure into the composite hydrogel makes there exist some non-covalent forces between the polymers, that is, the method of physical and chemical double cross-linking can effectively improve the mechanical strength of the hydrogel (Zhao et al., 2016; Zhang et al., 2019a). It can also make the hydrogel have certain self-healing property (Ai et al., 2021). Li N. et al. (2020) designed a hemicellulose hydrogel with high toughness by combining the chain expansion reaction of xylan with semi-IPN structure. Xylan was successfully modified to overcome the main limitation of brittleness through reductive amination reaction. Firstly, a cross-linking agent is added to construct a chemical cross-linking network of chain extended xylan (CEX). Then Fe^3+^ are introduced into it, and a physical cross-linking network is constructed by metal-ligand interactions. The hydrogel prepared by this method shows excellent mechanical properties and good water absorbency. And the water absorption and electrical conductivity can be adjusted by changing the mole fraction of metal ions. This method provides a way to prepare xylan hydrogels with high mechanical properties. Han et al. (2022) mixed hemicellulose, polyvinyl alcohol (PVA) and non-toxic sodium trimetaphosphate (STMP) crosslinker, heated and stirred, and formed a semi-interpenetrating structure by physical and chemical cross-linking. PVA contains rich hydroxyl groups that react with ring opened STMP to form a chemical cross-linking network. Xylan macromolecules do not react with PVA or STMP, but form a physical network connected by hydrogen bonds with the PVA-STMP chemical cross-linking system. This is a safe and facile method, and the hydrogel prepared shows high compressive strength (84.2 MPa at fracture strain of 90%), excellent compressive resilience, and thermal stability (showing a degradation temperature between 350 and 370°C). Moreover, due to its good biocompatibility and non-toxicity, it offers a great potential application in the field of biomedicine.

In the future, the mechanism of composite hydrogels should be studied comprehensively and deeply, so as to regulate its performance and expand its application range.

## 3 The applications of hemicellulose-based hydrogels

Hemicellulose-based hydrogels can respond to temperature (Liu et al., 2017a), PH (Zhao et al., 2014a), electricity (Zhao et al., 2014b), magnetism (Li et al., 2014), light (Cao et al., 2014) and other stimuli, and have a wide range of applications in drug delivery, bone tissue engineering, wound dressing, sewage purification, flexible electronic sensor and other fields (Kong et al., 2018).

### 3.1 Biomedical material

Hemicellulose has good biological activity, so it has broad application prospects in the field of biomedicine. It has been reported that xylan hemicellulose has the effect of inhibiting cell mutation, and has the function of detoxification and anti-inflammation, which greatly broadens the application of xylan in the field of biomedicine (Oliveira et al., 2010; Chang et al., 2021). Drugs and polymer solutions are mixed and injected into the human body and gelatinized under certain physiological conditions. Drugs can be released at a specific time and location through hydrogel decomposition or other means (Gupta et al., 2002; Yan et al., 2010). Some functional molecules such as PAA, poly (N-isopropylacrylamide) (Gao et al., 2016), Fe_3_O_4_ (Zhao et al., 2015) *etc.* And hemicellulose are used together to prepare intelligent hydrogels, which can be applied to drug delivery.

**FIGURE 2:**
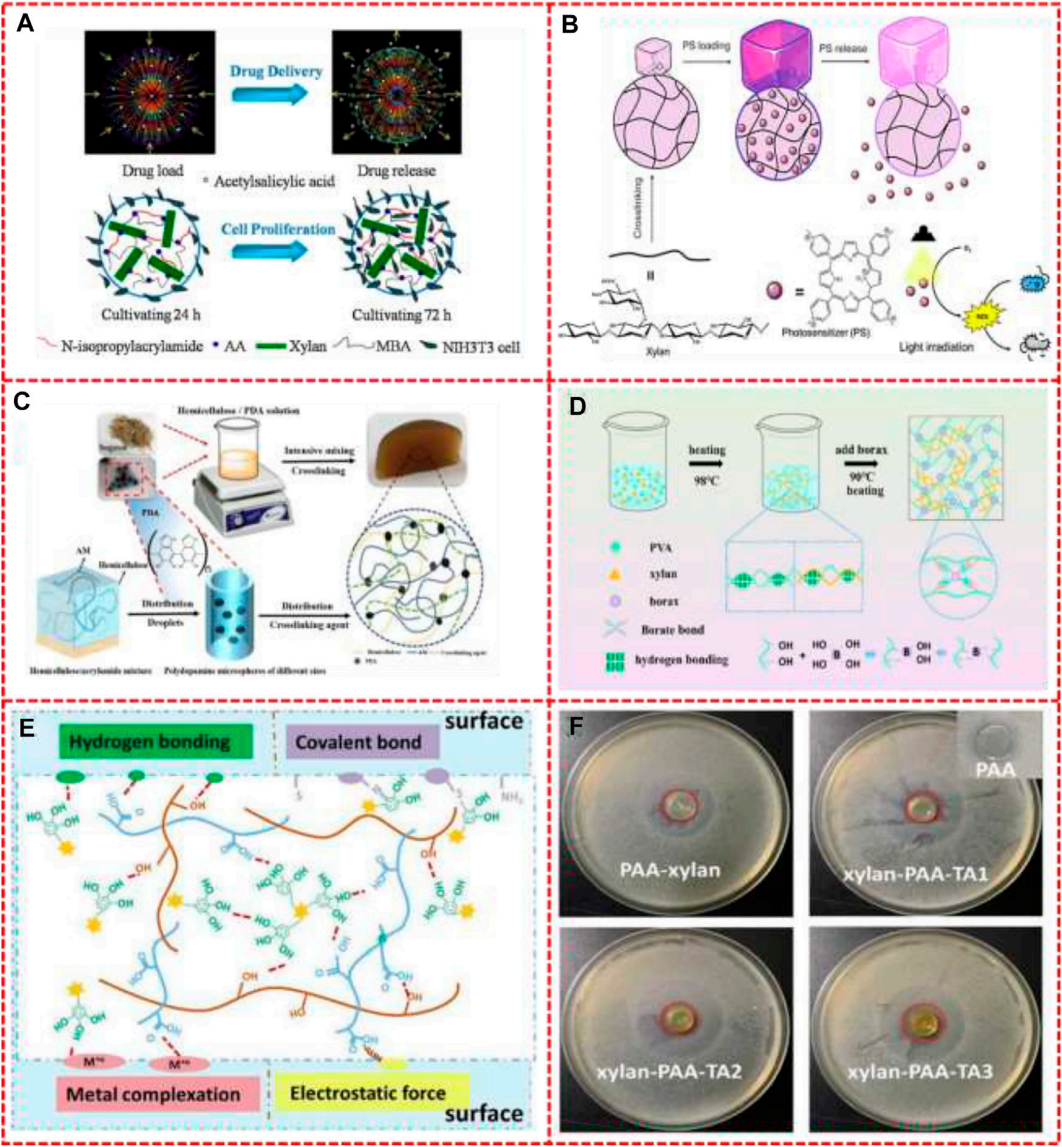
**(A)** The diagram of drug delivery behaviors and cell proliferation of the xylan-based P(NIPAm-g-AA) hydrogels copolymer networks, **(B)** The strategy of formation of hydrogels and photodynamic antimicrobial chemotherapy, **(C)** The synthetic route of hemicellulose/PDA hydrogel, **(D)** Schematic illustration of the preparation process of xylan/PVA/B DN hydrogel and possible reversible diol-borate ester bonds, **(E)** The adhesion mechanism of xylan-PAA-TA hydrogels, **(F)** Photographs of the inhibition zone of the PAA, PAA-xylan hydrogel, xylan-PAA-TA1, xylan-PAA-TA2 and xylan-PAA-TA3 hydrogels, **(F)** Photographs of the inhibition zone of the PAA, PAA-xylan hydrogel, xylan-PAA-TA1, xylan-PAA-TA2 and xylan-PAA-TA3 hydrogels. Reproduced from: **(A)** Gao et al. (2016), Elsevier; **(B)** Elkihel et al. (2021), ACS Publications; **(C)** Ge et al. (2021), MDPI; **(D)** Ai et al. (2021), Elsevier **(E)** Chang et al. (2021), Springer; **(F)** Chang et al. (2021), Springer.

Figure 2 shows some effects of hemicellulose based hydrogels on controlled release of drugs and antibacterial activities. Gao et al. (2016) prepared xylan-based temperature/pH sensitive hydrogels by the crosslinking copolymerization of xylan with NIPAm and AA using MBA as a cross-linker and 2,2-dimethoxy-2-phenylacetophenone as a photoinitiator *via* ultraviolet irradiation. Then they tested the hydrogels for drug encapsulation and release and for its biological activity on cells. The drug loaded and controlled release behaviors and the diagram of cell proliferation of the xylan-based P(NIPAm-g-AA) hydrogels networks were illustrated in Figure 2A. The porous structure appeared during drug release. And the porosity of hydrogels permits loading of drugs into the hydrogel matrix and subsequent drug-controlled release at a rate dependent on the diffusion coefficient of the small molecule or macromolecule through the gel network. After the cells were cultivated in hydrogel, the number of cells increased significantly after 72 h compared with 24 h. Photodynamic antimicrobial chemotherapy or PACT has been shown to be a promising antibacterial treatment that could overcome the challenge of multidrug-resistant bacteria; Elkihel et al. (2021) report a simple strategy to synthesize a cross-linked hydrogel from beech xylan as shown in Figure 2B. The 5, 10, 15, 20-tetrakis (1-methylpyridinium-4-yl)-porphyrin tetraiodide (TMPyP) was chosen as a model of hydrophilic photosensitizer (PS) and was encapsulated inside the xylan-based hydrogel. The positively charged ammonium groups of PS will allow the TMPyP to make ionic bonds with the carboxyl groups of the hydrogel, which facilitates its encapsulation. The hydrogel showed good properties of high swelling ratio, interconnected porous structure, and good mechanical integrity. TMPyP-loaded hydrogel prolonged release of PS up to 24 h with a cumulative amount that could reach 100%. TMPyP-loaded hydrogel showed a photocytotoxic effect against *Pseudomonas aeruginosa*, *Escherichia coli*, *Staphylococcus aureus* strains, and *Bacillus cereus*, while no cytotoxicity was observed in the dark and a strong potential for photodynamic antimicrobial chemotherapy. Ge et al. (2021) prepared hemicellulose-based composite hydrogels by adding polydopamine (PDA) microsphere as reinforcing agents (the synthetic route was showed in Figure 2C). Hemicellulose was extracted from bagasse and mixed with dopamine microspheres to form hydrogel under the action of crosslinking agent. The gaps in the network are filled with PDA and increased the density of the hydrogel. The hemicellulose-based hydrogel has an obvious drug release effect, and the release behavior of the composite hydrogel shows more obvious pH responsiveness. The result showed that complex hydrogels, delivered orally to the stomach as drug carriers, can reduce swelling of the gels and inhibit drug release under acidic conditions. As shown in Figure 2D, the xylan/borax/PVA double-network hydrogels were fabricated by Ai et al. (2021). The first physically and chemical network formed *via* numerous hydrogen bonds from PVA associated xylan and borax−PVA complexation between hydroxyl groups and borax. Then the hydrogel was subjected to a freeze-thaw process to form the second physically cross-linked network through the generation of the PVA crystal domain. The complexation between the tetrafunctional borate ion and the hydroxyl group of PVA is reversible resulting in the self-healing properties of hydrogels. The network between xylan and PVA and PVA crystal domain will provide a stable structure to withstand stress. Therefore, the hydrogels show nice strength, toughness, and stability. As shown in Figure 2E, the light blue rectangle meant the surface of different materials. There were several kinds of interaction between the hydrogel and different surfaces including hydrogen bonding, metal complexation, electrostatic force, and some kinds of covalent bond. And as shown in Figure 2F, for PAA hydrogel (control sample), there was no obvious inhibition zone in the disk. For PAA-xylan hydrogel, only a small inhibition zone was observed, which might be due to the antibacterial properties of xylan. In contrast, the xylan-PAA-TA hydrogels has the larger inhibition zone.

Modifications of hemicelluloses can improve their drug release and encapsulation properties of the prepared hydrogels. Gao et al. (2015) compared the acetylsalicylic acid release properties and biological activities of xylan and glycidyl methacrylate-modified xylan (GMAX) based hydrogels. The introduction of GMAX makes the pore size of hydrogels smaller and the network of hydrogels more uniform and orderly, so GMAX-Gels have a stronger electrostatic effect on acetylsalicylic acid, while xylan hydrogels have a low electrostatic interaction with acetylsalicylic acid due to the irregular internal structure. Compared with xylan hydrogel, GMAX hydrogel has higher cumulative drug release (84.2%) and longer release time in intestinal fluid, and also has higher encapsulation efficiency (95.21%). Because of their low release rate in gastric fluid and good cell compatibility, they can alleviate the side effects of long-term use of acetylsalicylic acid in patients.

Kong et al. (2017) prepared a novel hydrogel with temperature/pH dual sensitivity good swelling–deswelling properties, and honeycomb-like architecture through polymerizing maleic anhydride modified xylan (MAHX) with NIPAm and acrylic acid under UV light. The degree of substitution MAHX could control the pore volume, the mechanical properties, and the drug release rate of the hydrogel. Besides, in the gastrointestinal sustained drug release, the acetylsalicylic acid release rate was extremely slow at initial 3 h in the gastric fluid (24.26%), and then the cumulative release rate reached 90.5% after sustained release for 5 h in the stimulated intestinal fluid. Importantly, MAHX-based hydrogels had satisfactory biocompatibility with NIH3T3 cells. Therefore, MAHX-based hydrogels as drug carriers have potential application in human drug-delivery fields. Liu et al. (2017b) synthesized a temperature- and pH-sensitive hydrogel from NIPAAm, IA, hemicellulose and PVA. IA is rich in hydrophilic groups (-COOH). The lower critical solution temperature (LCST), the network dimension and equilibrium swelling rate of hydrogels can be adjusted by IA content. The LCSTs and equilibrium swelling ratio of the hydrogels increased with the increase of IA dosage in hydrogels. The LCST of the hydrogel could be adjusted to around body temperature by manipulating mass ratio of NIPAAm to IA 96/4. The carboxyl-carboxyl hydrogen bonding, existing between carboxyl in IA and carboxyl in salicylic acid, is in favor of the drug loading capacities of the hydrogels. The controlled release properties make the hydrogels as possible carriers in terms of controlled drug release. Gami et al. (2020) synthesized xylan-β-cyclodextrin hydrogel (XCD) by chemical crosslinking method and studied the absorption and release properties of the hydrogel on curcumin and 5-fluorouracil (FU) *in vitro*. When the hydrogels were in PBS, the drugs on the surface of the gel can be released immediately, so the initial release rate is faster. Curcumin could form inclusion complexes with βCD in the gel to prevent its release. XCD5 gel has a lower βCD content, so it has a better release effect on curcumin. The absorption of XCD1 to 5-FU was better because of the high content of βcd, which can provide more binding sites for 5-FU and promote the release of XCD1. After 24 h, the release of 5-FU and curcumin in PBS was 56% and 37%, which showed a favorable potential in the field of controlled drug release. Wang et al. (2022b) introduced BaGNP into the GGMMA/CNC-SH-based injectable hydrogel prepared through light-induced thiol-ene reaction. BaGNP is a kind of nano-filler with good biocompatibility. When combined with hydrogel, BaGNP is known to be a nano-filler with good biocompatibility. Combined with hydrogel, BAGNP can be used as a delivery system for some therapeutic ions, such as Si, Ca, or/and Cu ions to be released in the human body. It has been shown that the GGMMA/CNC-SH/BaGNP hydrogel can achieve sustained release of Si and Ca ions/species.

In addition, the combination of xylan and chitosan has superior antioxidant, antibacterial and cell proliferation ability than its parent polymer, and the hydrogel formed by the combination of hemicellulose and chitosan has long-term biocompatibility (Ali et al., 2020). Studies have shown that xylose hydrogels can be applied to injectable biological materials and contribute to fracture healing (Bush et al., 2016). Ali et al. (2022) introduced hydroxyapatite (HAP), whose chemical composition is similar to inorganic composition of bone, and oxide/reduced graphene oxide (GO/RGO), which has the advantages of enhancing cell response and regulating protein adsorption and cell behavior, into hemicellulose chitosan hydrogel. The pore size of the hydrogel samples containing GO/RGO came to be between 100 and 200 μm, which is beneficial for nutrient and gas exchange, angiogenesis and osteoinduction in human body. Due to the variety of species on GO, the addition of GO can improve the adsorption efficiency of composite hydrogels for proteins. The addition of GO also improved the mineralization tendency of deposited apatite and showed good osteogenic capability. The synergistic effect of HAP and GO together improves the ALP activity of the composite hydrogel, making it a potential application in bone tissue engineering.

The flexibility and biocompatibility of hemicellulose-based hydrogels make them also useful as nanofibrous scaffolds for the application of cardiac tissue engineering. Venugopal et al. (2013) prepared a nanofiber scaffold formed by crosslinking xylan/polyvinyl alcohol and glutaraldehyde vapor and conducted experiments in rat neonatal cardiomyocytes. The result shows that the nanofiber scaffolds can enhance the proliferation of cardiomyocytes and can be used in cardiac tissue engineering.

Hemicellulose dressings have been shown to be able to treat various skin lesions or skin diseases and are a safe and effective wound dressing (Melandri et al., 2006). Liu et al. (2016) composed polysaccharide composite hydrogels with different types of hemicellulose and nanocellulose. Then the morphology, mechanical strength and cell compatibility of the composite hydrogel were changed by changing the type, amount and way of hemicellulose incorporation. The performance of the hydrogel scaffold can be evaluated by observing the proliferation of 3T3 fibroblasts in the composite hydrogel. Therefore, this polysaccharide composite hydrogel has the potential to support cell growth and proliferation and can be used in wound healing. The Xylan-tyramine composite hydrogel prepared by Kuzmenko et al. (2014) through enzymatic cross-linking showed good mechanical property and high swelling property. The hydrogel showed the ability to differentiate into adipocytes in cell differentiation assays and could be used to encapsulate mesenchymal stem cells. Markstedt et al. (2017) prepared ACGGM-based hydrogels in the initiator system of hydrogen peroxide/horseradish peroxidase. In the cytotoxicity test, the cell survival rate of hydrogel was 95%, indicating that it had no cytotoxicity, which may be used in cell encapsulation, cell delivery and 3D cell culture.

Gelatin is a low-cost, non-toxic, and biodegradable natural collagen polymer, which is able to improve its properties by chemical cross-linking due to its large number of functional side groups, thus preparing a variety of stable materials. Synthetic polymers as crosslinkers inevitably cause a decrease in biocompatibility, but the combination with xylan solves these problems. Fu et al. (2020) directly oxidized xylan to dialdehyde xylan and used it as a cross-linking agent of gelatin to prepare hydrogel. Glycerol (Gly) and nicotinamide (NCA) were further introduced into the gel, and the *in vitro* release, Antibacterial activity and cytocompatibility test were conducted. NCA diffused rapidly in the resultant hydrogels, releasing them faster than most bio-based hydrogels. The resultant hydrogel had obvious inhibitory effect on the growth of yeast, *Bacillus subtilis* and *Staphylococcus aureus*, and has good viability and cell cytocompatibility. The results show that the hydrogel has a good application prospect in skin care.

In conclusion, Hemicelluloses-based hydrogels have been widely used in the field of medical materials due to their biological activity, controllable swelling, and mechanical properties. In addition, it can be combined with some organic or inorganic particles to further expand its range of applications, including controlled release of drugs, wound dressings, bone tissue engineering, even cardiac tissue engineering and facial care. However, most of the research still exists in the laboratory stage and needs to be scaled up in future studies to really make a difference.

### 3.2 Industrial adsorption

Nowadays, environmental pollution has attracted global attention. The pollution of water resources caused by heavy metal ions released by industry has become one of the most serious problems. Traditional methods for removing heavy metal ions from aqueous solutions have some disadvantages, such as high cost, low efficiency and other wastes (Ahluwalia and Goyal, 2005). Bioadsorption refers to the adsorption of heavy metals by organisms and their derivatives on water. It is a method with high efficiency, repeatability and other advantages, which is an alternative to traditional adsorption methods (Peng et al., 2012). The swelling of hydrogel increases its surface area, and some solute molecules interact with hydroxyl, carboxyl and amine groups in hydrogels through ionic and covalent bonds, so that hydrogels can be used as adsorbents (Seera et al., 2021). The chemical composition of hemicellulose and the network structure of hydrogel are the fundamental factors affecting the adsorption performance of hemicellulose hydrogel. Hemicellulose is easily hydrolyzed by acid, so it cannot be used in highly acidic environment (Xiang et al., 2022). Studies have shown that hemicellulose-based hydrogels as adsorbents have good adsorption effects on metal ions such as Pb^2+^, Cd^2+^, Cu^2+^, and Zn^2+^.

The addition of acrylamide, acrylic acid and other synthetic polymers into hemicellulose hydrogels can also improve the adsorption performance of hydrogels (Peng et al., 2012; Zhong et al., 2013). AA, an unsaturated carboxylic acid with pH sensitivity, is a common hydrogel additive that can be used not only to adsorb heavy metal ions but also polymer dyes. Xylan and AA graft copolymerized hydrogels showed good repeatability in plenty of adsorption/desorption cycles (Peng et al., 2012; Xu et al., 2019). In addition, the introduction of some adsorbable materials, such as chitosan (Shu-Ping et al., 2017), montmorillonite (MMT) (Zhang et al., 2014) and Fe_3_O_4_ (Sun et al., 2015), can also improve the adsorption efficiency of hydrogels. Shan et al. (2021) prepared environment-friendly hydrogels with cellulose, hemicellulose and lignin extracted from straw. Cellulose fibrils had crosslinked with lignin and hemicellulose by PAA chains in the presence of MBA. The resultant hydrogel had higher swelling rate, which was conducive to the diffusion of metal ions in the hydrogel, so the adsorption performance was improved. The adsorption behavior of straw biopolymer-based hydrogel was the result of ion exchange, which was spontaneous and reversible, and the prepared hydrogel adsorbent had good performance. The experimental results show that the hydrogel has good removal rate for heavy metal ions and water retention performance and has potential applications in water treatment and soil remediation. Kundu et al. (2019) prepared a composite hydrogel from xylan and β-cyclodextrin (βCD), capable of adsorption of Cd^2+^ and Ni^2+^, using ethylene glycol glyceride as cross-linking agent. The hydrogels had better adsorption abilities toward Cd II) than Ni II). This is a strategy to enhance the adsorption performance by enriching hydroxyl groups in hydrogels. Studies have shown that hydroxyl groups have strong affinity for heavy metal ions, so increasing the content of carboxyl groups in hemicellulose hydrogels is another strategy to improve the adsorption performance of hydrogels (Qu et al., 2017).

**FIGURE 3:**
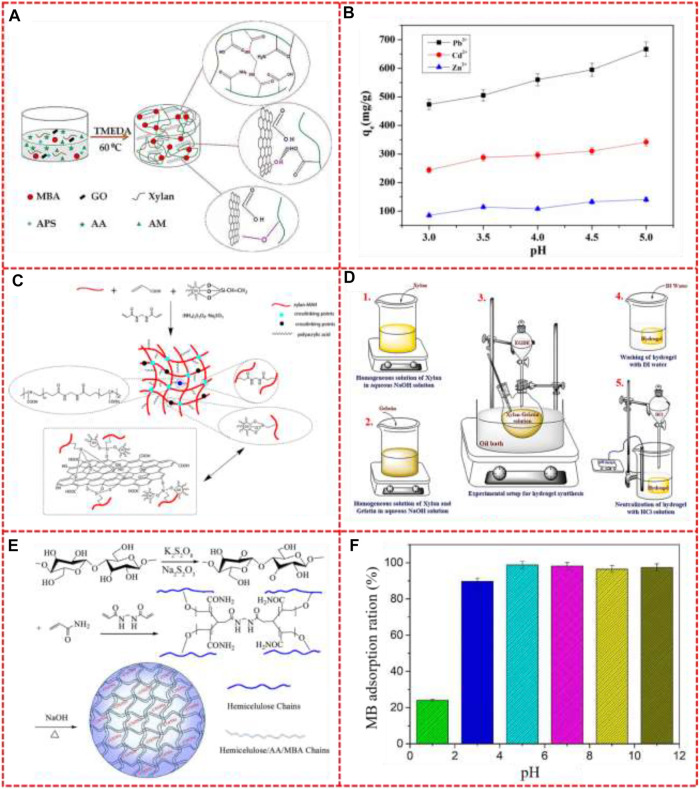
**(A)** The preparation process of xylan-g-/P (AA-co-AM)/GO nanocomposite hydrogels, **(B)** The influence of pH on the adsorption property of xylan-g-/P (AA-co-AM)/GO nanocomposite hydrogel, **(C)** Synthetic scheme of the chemically crosslinked xylan/GO composite hydrogel, **(D)** Synthesis procedure in the preparation of xylan and gelatin-based hydrogel, **(E)** The mechanism for the synthesis of the porous EIHs-g-PAA hydrogels, **(F)** Adsorption ratio for 250 mg/L of MB solution with different pH. Reproduced from: **(A)** Kong et al. (2019), MDPI; **(B)** Kong et al. (2019). MDPI; **(C)** Sun et al. (2022), Springer; **(D)** Seera et al. (2021), Elsevier; **(E)** Hu et al. (2022), Taylor & Francis Online; **(F)** Hu et al. (2022), Taylor & Francis Online.

Figure 3 shows the preparation and adsorption effect of hemicellulose-based hydrogel with wastewater adsorption. Xylan-g-/P (AA-co-AM)/Graphene oxide (GO) hydrogels were prepared by Kong et al. (2019) for the adsorption of metal ions. A large number of hydroxyl groups in the xylan chain react with ammonium persulfate (APS) to produce a large number of free radicals, which triggers the polymerization of acrylic acid (AA), acrylamide (AM) and GO sheets on the xylan branch chains. The active functional groups of hydroxyl and carboxyl in GO are polymerized through indirect branches of the polymer chain to form covalent or hydrogen bonds. Xylan/GO/P (AA-co-AM) hydrogels formed complex networks under the action of the crosslinking agent MBA, as shown in Figure 3A. Figure 3B illustrates the influence of pH on the adsorption property of hydrogels. With the increase of pH, the adsorption capacity of Pb^2+^ Cd^2+^ Zn^2+^ by hydrogel increases. When pH is low, protonation of active functional groups in hydrogel reduces the adsorption binding site of heavy metal ions and hinders adsorption. With the increase of pH, protonation gradually weakened, more active groups and metal ions chelate, improve the adsorption capacity. Sun et al. (2022) prepared composite hydrogel based on acylated xylan and silanized graphene oxide *via* free radical polymerization as a novel adsorbent for the removal of Cu^2+^ ions from aqueous solution. The synthetic scheme is described as Figure 3C, the carbon-carbon double bond was first introduced into xylan and GO, and then the hydrogel was polymerized with AA under the action of crosslinking agent. An ideal reusability of the hydrogel was also obtained and showed good potential in the removal of heavy metal ions in wastewater.

In the wastewater discharged by leather, textile, printing and other industries, there are toxic substances such as refractory dyes that threaten the water quality of the ocean and the living things in it (Ren et al., 2018). Methylene blue is a common dye in these industries. The presence of methylene blue in aqueous solution is usually cationic and electrostatic attraction is the main factor of ionic dye adsorption, so hemicellulose adsorbent containing anions has a good adsorption effect on it. Gelatin is composed of protein (90%–92%), mineral salts and water. Gelatin molecules can be used as adsorbents for toxic substances because of its large number of multifunctional groups (Thakur et al., 2017). Seera et al. (2021) synthesized xylan/gelatin composite hydrogels and studied their adsorption of methylene blue. Firstly, Xylan and gelatin were mixed in aqueous NaOH solution and stirred at 65°C (steps 1 and 2 of Figure 3D). The precursor solution was then transferred to a round-bottled flask and stored in an oil bath of 50°C. The crosslinking agent EGDE was then dropped for 30–40 min until the gel formation was visually confirmed (step 3). Afterward, hydrogels were scrupulously washed with DI water and neutralized with .1 M HCl to remove the excess number of precursors and NaOH (steps 4 and 5). The binding sites formed by the crosslinking of gelatin and xylan have electrostatic interaction with dye molecules. When the ratio of xylan to gelatin in hydrogel is 87:13 mol%, the adsorption efficiency of methylene blue is the highest.

Hu et al. (2022) prepared hydrogels based on hemicellulose from cold caustic extraction wastewater for efficiently adsorbing methylene blue. The mechanism for the synthesis of the EIHs-g-PAA hydrogels was shown in Figure 3E. When enough hydroxyl groups in the hemicellulose chains are activated to form hydroxyl radicals, the carbon-carbon double bond in Am reacts with hydroxyl radicals and MBA to form a massive gel material with a network structure in Na_2_S_2_O_3_/K_2_S_2_O_8_ oxidation–reduction catalysis system. And the resultant hydrogel has been evaluated at different pH (the result shows in Figure 3F). It can be seen that for a 250 mg/L MB solution, the removal rate of MB from the hydrogel increases from 24% to 89% as the PH increases from 1.0 to 3.0. When PH is higher than 3.0, -COOH, which has weak binding ability with positive charge MB molecule, starts to transform into -COO-, which has strong binding ability with MB molecule. Therefore, when pH value at 4.0, the adsorption capacity of the gel to MB was larger (98%). In addition, the electrostatic repulsion between -coo-groups enlarges the network structure of the hydrogel and promotes the diffusion of MB molecules into the hydrogel for adsorption. Moreover, the negative charge on the surface of inorganic nanoparticles can provide active sites to enhance the interaction between cationic dye molecules and hydrogels, and inorganic particles can also improve their mechanical properties.

Cheng et al. (2016) prepared composite hydrogels from hemicellulose in corn stover under mild alkaline conditions and introduced clay nanosheets. The addition of clay can improve the mechanical strength of hemicellulose hydrogels, and the adsorption rate mainly depends on the number of active sites on the surface of the hydrogel, so the negative charge on the surface of clay nanoparticles provides more active sites, thus improving the adsorption capacity of methylene blue on hydrogels. Zhao et al. (2020) extracted hemicelluloses from corn cob, added acrylic acid and acrylamide, and introduced Fe_3_O_4_ through *in situ* precipitation method, successfully prepared hemicelluloses based magnetic hydrogel. The Fe_3_O_4_ can increase the adsorption sites of methylene blue in the composite hydrogel, so that it shows good adsorption performance to methylene blue solution, which has broad application prospects in the field of industrial dye wastewater treatment.

In general, the hemicellulose hydrogels can adsorb metal ions or dyes in wastewater either by copolymerizing with other adsorptive polymers or by introducing other particles. On the one hand, the bioadsorption capacity of hemicellulose-based hydrogels is attractive and has potential in the field of industrial wastewater treatment. On the other hand, compared with other hydrogels, it is still in a relatively backward stage and cannot be applied on a large scale. The hemicellulose-based adsorbent hydrogels will provide potential choice for heavy metal ions and dyes removal.

### 3.3 Sensor

To realize the preparation of green, environmentally friendly, and non-toxic hydrogel sensor, biomass resources can be used as the skeleton or additive of hydrogel to control the performance of the sensor. Recent studies have shown that adding cellulose (Tong et al., 2019) or lignin (Wang et al., 2019a) as additives to hydrogels can modify their properties and enable them to be used in flexible strain sensors. As one of the biomass resource, hemicellulose is also a good choice in the field of flexible strain sensor.

**FIGURE 4:**
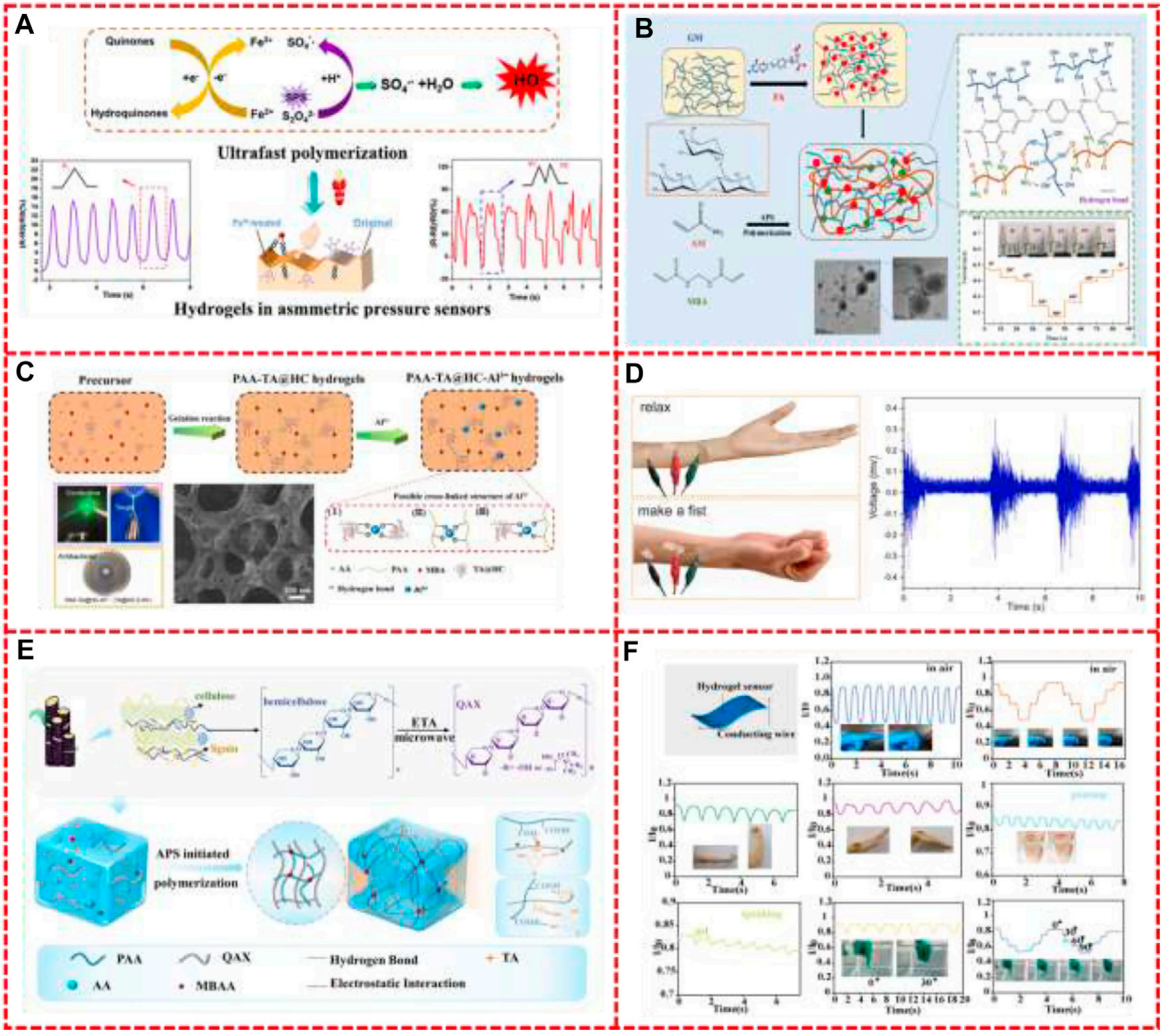
**(A)** Mechanism and pressure sensor signals recorded of PAA/TA@HC/Fe^3+^ hydrogels with or without Fe^3+^ treatment, **(B)** Schematic illustration of the preparation process of GM hydrogels and monitor arm bending as a strain sensor, **(C)** A schematic diagram of the ionic PAA-TA@HC-Al^3+^ hydrogel synthesis process and mechanism, **(D)** PAA-TA@HC-Al^3+^ hydrogel acts as a self-adhesive sensor, which can EMG signals, **(E)** Fabrication of the PAA-QAX-TA hydrogels, **(F)** PAA-QAX-TA hydrogel-based sensor model and sensing performance test, Reproduced from: **(A)** Gong et al. (2022b), ACS; **(B)** Ling et al. (2022), Elsevier; **(C)** Gong et al. (2022a), Elsevier; **(D)** Gong et al. (2022a), Elsevier; **(E)** Chang et al. (2022), Elsevier; **(F)** Chang et al. (2022), Elsevier.

Figure 4 shows the synthetic mechanism and sensing properties of some hemicellulose based hydrogels. As Figure 4A, the resistivity of the PAA-TA@HC-Al^3+^ hydrogel prepared by Gong et al. (2022a) decreased as the strain increased, because of the weakening of its conductive network (Zhang et al., 2021c). This hydrogel sensor can detect the movement on stretching, finger bending and weak pulse on the wrist. And the hydrogel sensor can detect throat vibration and it can also recognize different frequencies of breathing and phrases. Moreover, the hydrogels can accurately detect the weak ECG and EMG signals when it was used as ionic hydrogel electrodes. Additionally, skin tension-induced electrical signals can also be detected as Figure 4D. They also selectively applied Fe^3+^ on the surface of the hydrogel to obtain hydrogels with different adhesive properties on the upper and lower surfaces (Figure 4A). The strong adhesive side adhered to the skin, while the low adhesive side was resistant to external pollutants and could be used as a pressure sensor, which has great potential in the field of wearable flexible sensors (Gong et al., 2022b).

Ling et al. (2022) combined polygalactomannose (GM) with folic acid (FA), introduced hydrogen bond connection, and then added initiator and crosslinker to polymerize with AM to form a double network structure, as shown in Figure 4B. Compared with ordinary hemicellulose hydrogel, the hydrogel exhibited better mechanical strength and good anti-fatigue properties. The results showed that FA improved the electrical conductivity of the composite hydrogel, and the hydrogel showed antibacterial properties with inhibition against more than 80% of *E. coli*. In addition, they sticked GM4 on the knee of a wooden doll. The knee was bending with increasing angles (0°, 30°, 45°, 60° and 80°), accompanied with stretching of the hydrogel sample. As detected, the current decreased with stepwise increased bending angle and can remain stable in each angle stage. The CMX-PAM hydrogel prepared by Li et al. (2021) has impressive mechanical properties, UV blocking and electrical conductivity, so it shows potential for applications in human motion sensors. The more CMX contents, the hydrogel network structure became tighter *via* the ion coordination (−COO− from CMX and Fe^3+^). This tight structure may hinder the path of conductive ions and increase the resistance of the sample. This hydrogel-based sensor can respond to the movements and detect the micro-bending angles of the neck, the finger and wrist. These movements can yield distinct response behaviors, and the resistance signals were fully recoverable and repeatable.

An underwater adhesive xylan-based hydrogel was prepared by Chang et al. (2022) through chemical and physical crosslinking. Firstly, xylan and ETA were mixed under microwave to obtain QAX. The hydrogel is then synthesized under the initiation of APS. The mechanism of fabrication is shown in Figure 4E, in which AA is polymerized into PAA and grafted to QAX through covalent bond, and hydrogen bond can be formed between TA and PAA or QAX. Moreover, due to its attractive adhesion ability, the hydrogel can be directly and tightly attached to the surface without additional fixation and can be used as a wearable soft strain sensor for monitoring human movement. The experimental results are shown in Figure 4F, when the hydrogel sensor is attached to the finger, elbow and wrist for periodic bending and stretching motion, it shows significant electrical changes, and also has the ability to distinguish different bending angles, with good repeatability.

Introducing conductive materials such as conductive polymers (Chen et al., 2019) or metal ions (Zhang et al., 2019b) into hydrogels is a common scheme to improve their sensing performance. PPY has excellent biocompatibility and conductivity, and as a conductive polymer, it can be used in wearable strain sensors (Kenry, 2018). Zhang et al. (2021b) prepared a hemicellulose/polypyrrole antifreeze conductive hydrogel, which has good strain sensitivity. By converting deformation into electrical signals, the relative resistance of the hydrogel rapidly increases with the tensile strain from 0% to 500%. The hydrogel can be used for flexible wearable sensors to detect the strain signals of human joints bending and stretching.

In summary, the current research on hemicellulose-based hydrogel sensors mainly focuses on wearable flexible sensors and medical signal sensors, and a lot of achievements have been made. In the future, more types of hemicellulose derivatives or some nanoparticles that can improve their properties should be developed to expand their application range and maintain their biocompatibility.

## 4 Conclusion and perspectives

Hemicellulose-based hydrogels demonstrate wide application prospects in medical materials, wastewater adsorption and sensors due to their renewable, good processability, environmental friendliness and low cost. Up to now, lots of research have been reported on raw material selection, crosslinking agent screening, physical and chemical crosslinking method, *etc.* However, there are still many problems to be solved.

(1) To improve the mechanical strength of hemicellulose hydrogels, toxic chemicals are inevitably used, which greatly limits their application. In the future, green and low energy consumption hemicellulose hydrogel polymerization methods need to be explored.
(2) The intelligent hemicellulose hydrogels with multiple responses should be widely concerned in order to have different applications in more fields.
(3) Most of the research on hemicellulose-based hydrogels are still in the laboratory stage. How to accelerate the large-scale application of hemicellulose-based hydrogels in sensing, wastewater treatment, biomedicine and other fields is another major problem.

It is believed that with the further research on hemicellulose-based hydrogels, these challenging problems will be solved. In conclusion, hemicellulose hydrogels have a certain position in the future, and we believe that through continuous in-depth research on them, they will become one of the substitutes for fossil-based materials.
